# Ribonucleotide Reductase Inhibition Triggers Ferroptosis in Genetically Defined Subsets of Non–Small Cell Lung Cancer

**DOI:** 10.1158/2767-9764.CRC-25-0829

**Published:** 2026-09-11

**Authors:** Utsav Sen, Dalia Natour, Subhamoy Chakraborty, Elisa Gobbini, Chih-wei Fan, Calvin Koelbel, Andrew Elliott, Ari M. Vanderwalde, Hossein Borghaei, Balazs Halmos, Deniz Demircioglu, Dan Hasson, Nishant Gandhi, Triparna Sen

**Affiliations:** 1 Department of Oncological Sciences, Icahn School of Medicine at Mount Sinai, New York, New York.; 2 Division of Medical Oncology, Department of Internal Medicine, The Ohio State University Comprehensive Cancer Center, Columbus, Ohio.; 3 Caris Life Sciences, Phoenix, Arizona.; 4 Department of Hematology and Oncology, Fox Chase Cancer Center, Philadelphia, Pennsylvania.; 5 Montefiore Medical Center, Albert Einstein College of Medicine, New York, New York.; 6 Tisch Cancer Institute, Icahn School of Medicine at Mount Sinai, New York, New York.; 7 Bioinformatics for Next Generation Sequencing (BiNGS) Core, Icahn School of Medicine at Mount Sinai, New York, New York.

## Abstract

Non–small cell lung cancer (NSCLC) is responsible for the majority of cancer-related mortality worldwide. Lung adenocarcinoma is the most common NSCLC subtype. Despite advances in targeted therapies, treatment resistance remains a critical challenge. Ribonucleotide reductase (RNR), a crucial enzyme in deoxyribonucleotide triphosphate biosynthesis, is frequently upregulated in cancer, contributing to genomic instability and poor prognosis in multiple malignancies. However, the role of the RNR complex in driving tumorigenesis is not fully understood in oncogene-driven lung adenocarcinoma. Transcriptomic analysis of more than 27,000 real-world samples of patients with NSCLC revealed that RNR subunits (*RRM1* and *RRM2*) are significantly upregulated in *TP53*-mutated NSCLC and are correlated with significantly poor prognosis in multiple oncogene-driven lung adenocarcinoma. Using pharmacologic and genetic approaches to inhibit RNR in lung adenocarcinoma models, we assessed functional consequences through molecular, biochemical, and imaging techniques. RNR inhibition induced appreciable replication stress and triggered DNA damage, leading to cell death in lung adenocarcinoma cells. Notably, we uncovered that RNR suppression preferentially induced ferroptosis, an iron-dependent cell death driven by lipid peroxidation. This represents a previously unrecognized mechanism of RNR-mediated cell death by which mutant lung adenocarcinoma cells can be selectively targeted. Our study establishes RNR inhibition as a potent strategy to selectively induce ferroptosis in oncogene-addicted lung adenocarcinoma, offering a new therapeutic avenue for genetically defined patient subgroups. Targeting nucleotide metabolism could serve as an effective approach to overcome treatment resistance and improve clinical outcomes for patients with high-risk lung adenocarcinoma.

**Significance:**

Our findings highlight RNR as a promising therapeutic target in oncogene-driven lung adenocarcinoma. By demonstrating that RNR inhibition induces ferroptosis, our study opens up new possibilities for developing targeted therapies that selectively eliminate cancer cells in lung adenocarcinoma, paving the way for personalized treatment strategies and potentially overcoming resistance to current therapies.

## Introduction

Non–small cell lung cancer (NSCLC) is a leading cause of cancer-related mortality worldwide (1). It represents significant challenges in modern oncology due to its high incidence rate, poor prognosis, and dismal survival rates associated with advanced-stage disease (2). Lung adenocarcinoma, the most common NSCLC subtype, is mainly driven by genetic alterations that can be used to predict patient prognosis and treatment strategies. The treatment landscape for advanced lung adenocarcinoma has rapidly evolved with the discovery of oncogenic drivers such as *KRAS*, *EGFR*, *ALK*, *MET*^ex^, *ROS1*, *RET*^+^, and others, enabling effective targeted therapies (3, 4). Despite impressive advances in recent years, metastatic aggressive lung adenocarcinoma remains a devastating disease, and treatment success is limited by the emergence of resistance (5).

As the therapeutic options expand, resistance mechanisms also become more common, leading to limited treatment strategies for relapsed disease. Given these challenges, developing novel therapies and rational combination approaches has become critical to overcoming resistance mechanisms, enhancing treatment durability, and improving patient outcomes in lung adenocarcinoma (6, 7). Targeting critical signaling pathways involved in cancer cell proliferation and DNA damage response is one of the most promising treatment approaches in cancer (8–12). Nucleotide metabolism, particularly ribonucleotide reductase (RNR), has emerged as a key mechanism in maintaining cellular homeostasis and the pathophysiology of malignant cells (13).

The RNR enzyme complex, mainly comprising two catalytic subunits, *RRM1* and *RRM2*, is crucial in maintaining the pool of deoxyribonucleotide triphosphates (dNTP), which are also critical for DNA replication and repair (14). Beyond its canonical role in dNTP synthesis, RNR integrates cell-cycle signals, particularly S-phase entry, and metabolic stress signals to sustain high proliferative demand in tumor cells (15). RRM2, in particular, is strongly induced by oncogenic KRAS, MYC, and E2F signaling, linking RNR activity to aggressive tumor phenotypes, genomic instability, and replication stress (16). RRM1 and RRM2 overexpression have also been implicated in chemoresistance, redox adaptation, and enhanced DNA damage tolerance, positioning the RNR complex as a central driver of tumorigenesis and therapeutic failure (17).

Recent studies have highlighted the upregulation of RNR activity in lung cancer (18–21). However, the role of *RRM1* and *RRM2* as biomarkers in the real-world setting and the underlying mechanisms by which *RRM1* and *RRM2* drive tumorigenesis, treatment resistance, and poor prognosis in lung adenocarcinoma are not fully understood.

In this study, we investigated the expression of *RRM1* and *RRM2* in 27,280 real-world NSCLC clinical samples and their correlation with prognosis. Our findings establish the role of RNR as a negative prognostic biomarker and potential therapeutic target. By establishing the mechanistic basis of RNR in lung adenocarcinoma, this study provides critical insights that support the development of RNR-targeted therapeutic strategies for oncogene-driven lung adenocarcinoma.

## Materials and Methods

### Next-generation sequencing of DNA (clinical data, methodology provided by Caris Life Sciences)

This retrospective study was conducted under Caris Life Sciences’ Research Data Banking protocol, which was reviewed and granted Institutional Review Board (IRB) exemption by the Western Copernicus Group IRB. The study adhered to the ethical guidelines of the Declaration of Helsinki, the Belmont Report, and the US Common Rule.

Between 2017 and 2022, Caris Life Sciences (Phoenix, AZ) molecularly profiled 27,280 NSCLC specimens for DNA and RNA sequencing. Next-generation sequencing was performed on genomic DNA isolated from formalin-fixed, paraffin-embedded (FFPE) tumor samples using the NextSeq or NovaSeq 6000 platform (Illumina, Inc.). For the NextSeq-sequenced tumors, a custom-designed SureSelect XT assay was used to enrich 592 whole-gene targets (Agilent Technologies). Additionally, for the NovaSeq-sequenced tumors, 719 clinically relevant genes were enriched at high coverage and read depth, along with another panel designed to enrich for >20,000 genes at lower read depth. All variants were detected with >99% confidence based on allele frequency and amplicon coverage, with an average sequencing depth of coverage of >500 and an analytic sensitivity to identify variants with a variant allele frequency of ≥5%. Genetic variants identified were interpreted by board-certified molecular geneticists and categorized as pathogenic (P), likely pathogenic (LP), variant of unknown significance, likely benign, or benign, according to the American College of Medical Genetics and Genomics standards. When assessing mutation frequencies of individual genes, P and LP were counted as mutations. The demographics of the study population are described in Supplementary Table S1A and S1B.

### Whole-transcriptome sequencing

FFPE specimens underwent pathology review to measure the percentage of tumor content and tumor size; a minimum of 10% tumor content in the area for microdissection was required to enable enrichment and extraction of tumor-specific RNA. The QIAGEN RNA FFPE tissue extraction kit was used for RNA extraction, and the RNA quality and quantity were determined using the Agilent TapeStation. Biotinylated RNA baits were hybridized to the synthesized and purified cDNA targets, and the bait–target complexes were amplified in a postcapture polymerase chain reaction (PCR). The Illumina NovaSeq 6500 was used to sequence the whole transcriptome from tumor samples to an average of 60M reads. Raw data were demultiplexed by the Illumina DRAGEN Bio-IT accelerator, trimmed, counted, removed of PCR duplicates, and aligned to the human reference genome hg19 by the STAR aligner. Transcripts per million molecules (TPM) were generated using the Salmon expression pipeline. Specimens were stratified into four groups based on the quartile expression of *RRM1* or *RRM2*.

*Anaplastic Lymphoma Kinase Fusion* (*ALK*^FUS^) tumors were defined based on positive immunohistochemical (IHC) staining or the presence of an ALK fusion in the molecular analysis, whereas Kirsten rat sarcoma viral oncogene homolog mutation (*KRAS*^MUT^) and epidermal growth factor receptor mutation (*EGFR*^MUT^) tumors were defined based on the presence of P/LP mutations in these genes. The fatty acid metabolism and ferroptosis pathway gene panel were log_2_(TPM + 1)-transformed and *z*-scored. The average *z*-score for all genes in a given gene set was compared across the study cohorts.

A Kruskal–Wallis test was performed to assess overall differences in RRM1 and RRM2 expression among the groups, followed by pairwise Mann–Whitney U tests. *P* values were adjusted for multiple hypothesis testing using the Benjamini–Hochberg method. Median values of RRM1/RRM2 expression across the respective groups are reported.

### Survival analysis

Real-world survival information was obtained from insurance claims data and calculated from the time of tissue biopsy to death or last contact for overall survival (OS). Median OS was calculated using Kaplan–Meier estimates, and *P* values were calculated using the log-rank test.

*χ* ^2^, Fisher exact, Mann–Whitney *U*, and *t* tests (with Welch correction) were used and adjusted for multiple hypothesis testing using the Benjamini–Hochberg method where appropriate. Statistical significance was considered when *q* < 0.05.

### The Cancer Genome Atlas dataset analysis

TCGA Lung Adenocarcinoma (TCGA-LUAD, PanCancer Atlas) data were accessed through cBioPortal for Cancer Genomics. mRNA expression levels were analyzed using RNA-Seq by Expectation-Maximization-normalized *z*-scores, calculated relative to the expression distribution of each gene within the TCGA-LUAD cohort. Clinical and genomic annotation data were included for multivariate survival analyses.

Violin plots were generated using the ggplot2 package (v3.4.2) to compare RRM1 and RRM2 expression between TP53 wild-type (*TP53*^WT^) and TP53-mutant samples in the TCGA-LUAD cohort. Violin plots depict the distribution of expression values using kernel density estimation, with the width representing data density. Embedded box plots indicate the median and IQR, with whiskers extending to 1.5× the IQR. Statistical differences between groups were assessed using the Wilcoxon rank-sum test.

Survival analyses were conducted using the TCGA-LUAD cohort to evaluate the association of RRM1 and RRM2 gene expression with OS and progression-free survival. Multivariate Cox proportional hazards regression models were fitted, adjusting for clinical stage and key oncogenic alterations in KRAS, EGFR, and ALK. Cancer stage was modeled as a categorical variable with stage I as the reference group. Somatic alterations in KRAS and EGFR, as well as ALK alterations (fusion/IHC), were included as binary variables (mutated vs. wild-type). Gene expression was modeled as a continuous variable. Hazard ratios with 95% confidence intervals and corresponding *P* values were reported. The analysis was performed using the survival package (v3.2-11) and the survminer (v0.4.9) package in R (v4.1.0).

### Pharmacologic inhibitors

Triapine (S7470), gemcitabine (S1714), and ferrostatin-1 (Fer-1, S7243) were purchased from Selleck Chemicals, and TAS-1553 (HY-150785) was purchased from MedChemExpress (see Supplementary Table S2).

### Cell lines

All lung adenocarcinoma cell lines were obtained from the American Type Culture Collection, maintained in Roswell Park Memorial Institute (RPMI) medium supplemented with 10% fetal bovine serum (FBS), and incubated at 37°C with 5% CO_2_. The following cell lines were used in this study: NCI-H358, H23, PC9, HCC827, H2228v2, and A925L. All cell lines underwent fingerprinting to confirm identity and were tested *Mycoplasma*-negative before experiments. Early passages (passage 3–10) were used for experiments (see Supplementary Table S3).

### Lentiviral production

Lentiviruses were produced using cotransfection of 293T cells with lentiviral backbone constructs and packaging vectors psPAX2 and pMD2.G, using Lipofectamine LTX Reagent with PLUS Reagent (Invitrogen). Media was replaced with bovine serum albumin (BSA)–rich media containing DMEM 16 to 18 hours after transfection, and the supernatant was collected 72 hours after transfection. The supernatant was centrifuged and collected to avoid any cell debris, and the aliquots were stored at −80°C.

### Lentiviral transduction

Cells were plated in 10-cm dishes at 10^6^ cells/dish on day 0 and were incubated overnight. On day 1, media was aspirated from cell plates and replaced with 10 mL of fresh RPMI supplemented with 10% FBS and 8 μg/mL polybrene (Tocris, R&D Systems, 7711). Lentivirus from −80°C was quick-thawed in a 37°C water bath and returned to ice. Lentivirus was added to cells in a dropwise manner in a volume determined by viral titration, and cell plates were incubated overnight at 37°C. On day 2, lentiviral media was aspirated from cell plates and replaced with fresh RPMI with 10% FBS. Cell selection began on day 3, when applicable.

### Cell viability

Briefly, 3,000 cells were seeded in a white 96-well plate (cat. #353296, Corning) for the CellTiter-Glo (CTG) assay. Cells were allowed to grow for at least 18 to 24 hours and were treated with the different drugs of our interest (triapine, gemcitabine, and TAS-1553). After incubation with the drugs (based on our experimental requirements), the CTG assay was performed using our previously published protocol (22). All commercial kits used in the study are detailed in Supplementary Table S4.

### BODIPY/C11 staining

In a six-well plate, 0.2 × 10^6^ cells per well were seeded and allowed to attach overnight. Cells were then treated with media containing the indicated drug dose or equivalent dimethyl sulfoxide (DMSO) vehicle dose and returned to incubation at 37°C. After the indicated time, the drug media was supplemented with 3 μmol/L of C11-BODIPY 581/591 Lipid Peroxidation Sensor (Invitrogen, D3861), and the assay was performed using our previously published protocol (22). Vehicle-treated cells were compared with drug-treated cells, with a shift in the fluorescein isothiocyanate curve to the right, indicating increased lipid peroxidation.

### Crystal violet staining

For crystal violet staining, the cells were plated in a flat-bottom 96-well plate (3,000 cells/well) or in a six-well plate (0.25 × 10^6^) and were allowed to grow for at least 24 hours. Then, the cells were treated with the respective drugs (either alone or in combinations) based on our experimental interests. After 72 to 96 hours of incubation, the media was discarded, and the cells were washed 3 times with phosphate-buffered saline (PBS) and were then stained with a 2.5% crystal violet staining solution containing methanol. After 30 minutes of incubation, the staining solution was discarded and washed 3 to 5 times with PBS; images were captured by the scanner. All commercial kits used in the study are detailed in Supplementary Table S4.

### Measuring the cellular glutathione levels

Total glutathione (GSH) levels in the cells before and after treatment with triapine (1.5 μmol/L, 72 hours) were determined using a Glutathione Colorimetric Detection Kit (EIAGSHC, Thermo Fisher Scientific). Cell lysates were prepared with 1 × 10^6^ cells, and the GSH assay was performed using manufacturer-provided protocols. All commercial kits used in the study are detailed in Supplementary Table S4.

### Thiobarbituric acid reactive substance assay for malondialdehyde estimation

To measure malondialdehyde (MDA), H358, PC9, and A925L cells were plated in 10-cm dishes and allowed to grow for 24 hours. After 24 hours, cells were treated with 1.5 μmol/L of triapine. After 72 hours of treatment, the cells were trypsinized and washed with cold PBS. For MDA measurement, 1 × 10^7^ cells were taken for each condition and measured using a Thiobarbituric Acid Reactive Substance Assay Kit (Cayman Chemical, No. 10009055) according to the manufacturer’s protocol (Supplementary Table S4).

### Immunoblotting

Total proteins were extracted with radioimmunoprecipitation assay lysis buffer (cat. #89900). The amount of extracted protein was estimated by the Bradford method for Western blot analysis. Western blotting was performed using our previously published protocol (20). The list of primary antibodies is in Supplementary Table S5. The bands were visualized by enhanced chemiluminescence (ECL; Pierce, Thermo Fisher Scientific or Clarity ECL, Bio-Rad) reagent with an iBright Imaging System (Thermo Fisher Scientific). Protein quantification was analyzed using ImageJ to measure band intensity for the raw chemiluminescent images. Band expression was normalized to the density of the loading control (vinculin or β-actin).

### Immunofluorescence and confocal microscopy

For immunofluorescence (IF) analysis, the cells were seeded in eight chambered slides. Then, the cells were treated with either vehicle DMSO or 1.5 μmol/L of triapine. After 72 hours of treatment, the media was removed, and the slides were washed with Dulbecco’s PBS (DPBS) and then blocked with 1% BSA for 45 minutes at 37°C, followed by two 5-minute PBS washes. The cells were then permeabilized with a weak hypotonic solution (0.075 mol/L KCl/0.9% saline, 1:9), incubated at 37°C for 5 minutes, and washed with DPBS twice. The cells were incubated with phospho-γ-H2AX, phospho-RPA32, and lamin B1 primary antibodies (1:300 dilution) overnight at 4°C in a humidified chamber. After incubation, the cells were washed with PBS twice for 5 minutes, followed by incubation with secondary antibodies (Alexa Fluor 488 rabbit monoclonal and Alexa Fluor 647 mouse monoclonal; 1:1,000 dilution) for 1 hour at 37°C in a humidified chamber, followed by washing with PBS twice for 5 minutes. The cells were then counterstained with DAPI (2 μg/mL) for 30 minutes at room temperature. The stained cells were imaged with a Leica STED 3 times with a 63× lens. Antibody details are available in Supplementary Table S5.

### Cell-cycle analysis

For cell-cycle analysis, 0.5 × 10^6^ cells were seeded in a six-well plate. After attachment, cell synchronization was performed by serum starvation for 6 hours, followed by triapine treatment (1.5 μmol/L) for 72 hours at 37°C and 5% CO_2_. Cells were harvested after treatment, washed twice with PBS (1×), and fixed in 70% ethanol at −20°C. The fixed cells were stained with FxCycle PI/RNase Staining Solution (Invitrogen, cat. #F10797) and incubated in the dark for 15 minutes. Cells were then acquired in the BD FACSCanto II Flow Cytometer.

## Results

### RNR is associated with poor prognosis in oncogene-driven NSCLC

To comprehensively assess *RRM1* and *RRM2* expressions in NSCLC, we analyzed a large real-world cohort of *N* = 27,280 clinical samples with genomic and transcriptomic information.

First, we analyzed the coexpression of *RRM1* and *RRM2* in NSCLC. We found a strong positive correlation between *RRM1* and *RRM2* in lung adenocarcinoma tumors (Supplementary Fig. S1A; *r* = 0.71; *P* < 0.0001), and this relationship remained significant across the most common oncogenic drivers such as *KRAS*^MUT^, *EGFR*^MUT^, and *ALK*^FUS^ subgroups (Supplementary Fig. S1B–S1D; *r* > 0.71; *P* < 0.0001).

**Figure 1.**
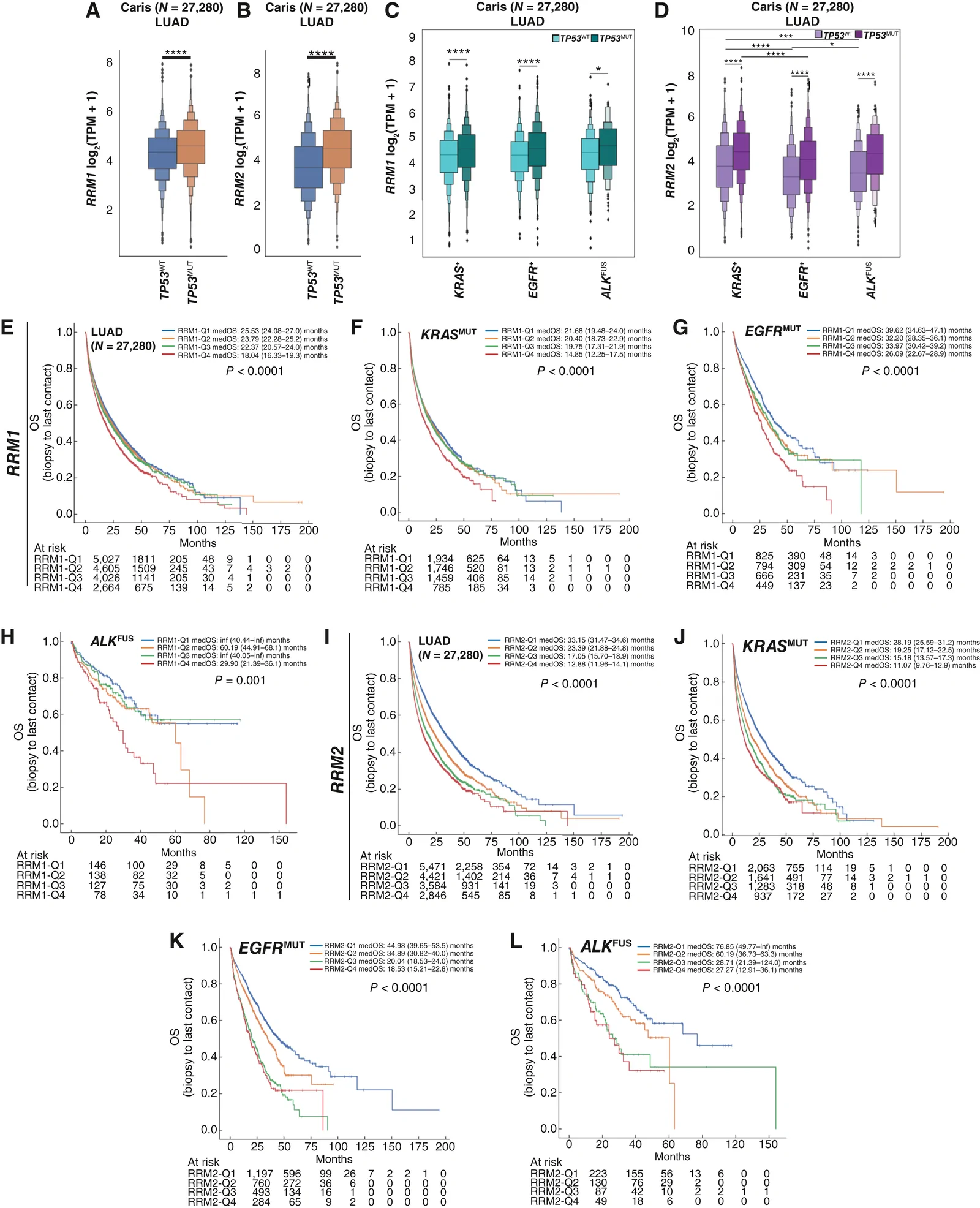
Higher levels of *RRM1* and *RRM2* expression are linked to worse prognosis in oncogene-driven NSCLC. **A,** Box plot representing the expression of *RRM1* and (**B**) *RRM2* in the clinical dataset of real-world NSCLC cases (*n* = 27,280; Caris data) according to the TP53 mutational status (*TP53*^WT^ and *TP53*^MUT^). **C,** Box plot representing the expression of *RRM1* and (**D**) *RRM2* in oncogene-addicted NSCLC, namely *KRAS*^MUT^, *EGFR*^MUT^, and *ALK*^FUS^ according to the TP53 mutational status. **E,** Kaplan–Meier plot according to the *RRM1* expression in the overall cohort. **F,***KRAS*^MUT^, (**G**) *EGFR*^MUT^, and (**H**) *ALK*^FUS^ populations. **I,** Kaplan–Meier plot according to the *RRM2* expression in the overall cohort; (**J**) *KRAS*^MUT^, (**K**) *EGFR*^MUT^, and (**L**) *ALK*^FUS^ populations. *χ*^2^, Fisher exact, Mann–Whitney *U*, and *t* tests (with Welch correction) were used and adjusted for multiple hypothesis testing using the Benjamini–Hochberg method where appropriate. Statistical significance was considered when *q* < 0.05. *, *P *< 0.05; ***, *P* < 0.001; ****, *P *< 0.0001 were considered as significant [*P *> 0.05 was considered as non-significant (ns)]. LUAD, lung adenocarcinoma; med OS, Median overall survival.

We then examined the mutational status of p53 and its potential impact on RNR function (Fig. 1A and B). Indeed, previous studies have shown that although WT p53 suppresses RNR activity primarily via mTORC1 inhibition, mutant p53 instead upregulates RNR expression and activity, thereby expanding nucleotide pools to support uncontrolled proliferation and driving its oncogenic gain of function (23, 24). Consistently, in our cohort, *TP53*-mutant (*TP53*^MUT^) tumors exhibited higher *RRM1* and *RRM2* expressions compared with *TP53*^WT^ NSCLC cases (Fig. 1A and B; Supplementary Table S6).

We then stratified cases by major driver mutations, including *KRAS* (*n* = 5,924), *EGFR* (*n* = 2,734), and *ALK* (*n* = 489) alterations, to evaluate their association with *TP53*, *RRM1*, and *RRM2* expression (Fig. 1C and D). Interestingly, co-occurring *TP53* mutation showed a significantly higher expression of *RRM1* (Fig. 1C; *P* < 0.001; Supplementary Table S7) and *RRM2* (Fig. 1D; *P* < 0.001; Supplementary Table S7) compared with *TP53*^WT^ across *KRAS*^MUT^, *EGFR*^MUT^, and *ALK*^FUS^ cases, suggesting an independent role of RNR in cancer biology. To independently verify our findings, we analyzed an available clinical TCGA-LUAD dataset in which we confirmed that *RRM1* and *RRM2* were significantly enriched in *TP53*-mutated lung adenocarcinoma cases compared with wild type (Supplementary Fig. S1E). We then examined the prognostic role of *RRM1* and *RRM2* expressions in NSCLC. High expression of *RRM1* was associated with poor prognosis in NSCLCs (Fig. 1E; *P* < 0.0001). *RRM1* expression also predicted significantly worse OS in *KRAS*^MUT^*EGFR*^MUT^, *ALK*^FUS^, and *MET*^ex14^ NSCLC (Fig. 1–H; Supplementary Fig. S1F; *P* < 0.0001). Similarly, *RRM2* expression predicted significantly poorer prognosis in NSCLCs (Fig. 1I; *P* < 0.0001) across *KRAS*^MUT^, *EGFR*^MUT^, and *ALK*^FUS^ and in *MET*^ex14^, *RET*^+^, and *ROS*^+^ (Fig. 1J–L; Supplementary Fig. S1G–S1I; *P* < 0.0001).

To address the potential influence of disease stage on the prognostic relevance of RNR subunits, we evaluated the TCGA dataset by evaluating OS with RRM1 and RRM2 correlation stratified by mutation subtype. Although both higher *RRM1* and *RRM2* expression increased the risk of death in cases by 20% and 30%, respectively, stage progression was far more impactful (Supplementary Figs. S2A–S2C and S3A–S3C). Stage II cases decreased the likelihood of survival by twofold, whereas it was at least threefold for both stage III and IV cases (Supplementary Figs. S2A–S2C and S3A–S3C). These findings were independent of either RNR expression or *KRAS*, *EGFR*, or *ALK* comutations. Collectively, our findings indicate that although both *RRM1* and *RRM2* exhibit stage-independent prognostic impact, the RNR complex as a whole (RRM1/RRM2) continues to show a clinical impact in both early and late disease, notably the strong effects observed in stage III and IV cases.

By leveraging a large, real-world clinical dataset, we provide a comprehensive analysis of *RRM1* and *RRM2* expressions in NSCLC. Our findings highlight that these key nucleotide synthesis genes are consistently upregulated in the presence of *TP53* mutations, regardless of the presence of oncogenic drivers. Importantly, high expression of *RRM1* and *RRM2* is associated with poor prognosis, reinforcing their potential as biomarkers and therapeutic targets in NSCLC.

### Targeting RRM1 and RRM2 impairs the viability of oncogene-driven lung adenocarcinoma

RNR is essential for DNA synthesis and dNTP production, making it a critical target in cancer therapy.

We previously reported a druggable genome library CRISPR screen in H358 (*KRAS* mutant) cells (12). Interestingly, the screen demonstrated that in H358, *RRM1* and *RRM2* were among the top depleted genes. This showed that *RRM1* and *RRM2* are essential genes required for the proliferation and survival of *KRAS*-mutant cells (Supplementary Fig. S4A; ref. 12).

We thus explored the effect of targeting RNR (by genetic and pharmacologic inhibition) in oncogene-addicted lung adenocarcinoma *in vitro* models. We investigated pharmacologic inhibition of RNR by treatment with three separate RNR inhibitors currently in either advanced clinical trials or in routine chemotherapy regimens (triapine, TAS1553, and gemcitabine) in a panel of different lung cancer cell lines. Our panel included *KRAS*-mutant (H358 and H23), *ALK*-mutant (H2228V2 and A925L), and *EGFR*-mutant (PC9 and HCC827) lung adenocarcinoma cell lines.

**Figure 2.**
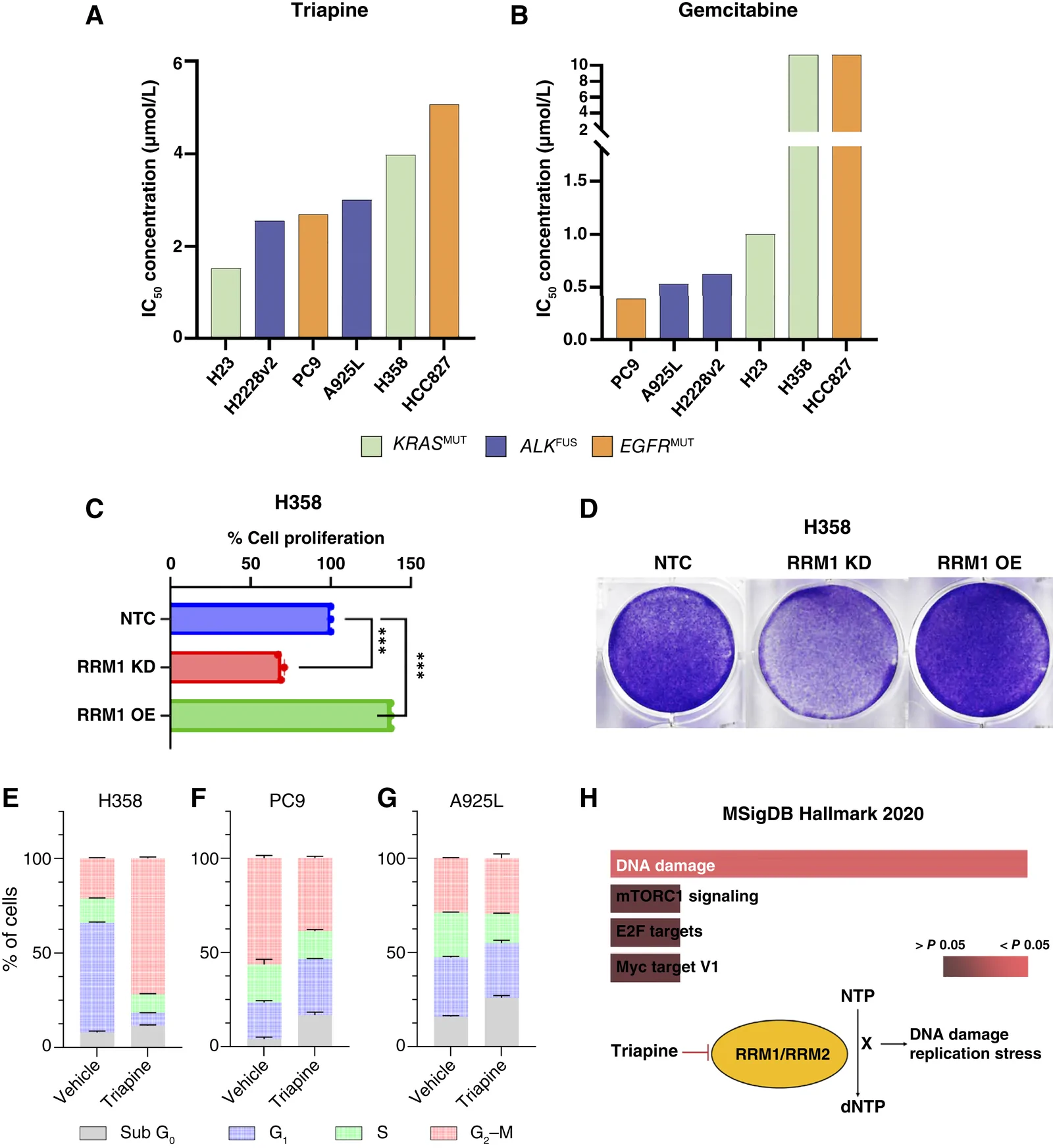
*RRM1* and *RRM2* inhibition reduces cell viability in oncogene-driven lung adenocarcinomas. Bar graphs representing the IC_50_ values of (**A**) triapine and (**B**) gemcitabine in different lung adenocarcinoma lines. **C** and **D,** Comparison of proliferation after genetic knockdown (KD) and overexpression (OE) of *RRM1* or non-targeting control (NTC) in H358 cells. Bar graphs represent cell-cycle phases upon triapine treatment (1.5 μmol/L, 72 hours) in different lung adenocarcinoma cell lines, namely (**E**) H358, (**F**) PC9, and (**G**) A925L. **H,** Representative pathway enrichment plot from the MSigDB database of genes associated with *RRM1* and *RRM2*. For comparisons among multiple groups, one-way analysis of variance (ANOVA) was performed; between two groups, a Student *t* test followed by Dunnett *post hoc* test was performed. ***, *P* < 0.001 were considered significant [*P* > 0.05 was considered nonsignificant (ns)]. The error bar represents the ± standard error of the mean (SEM), and the results are a representation of *n* = either 2 or 3 based on the experiments.

Lung adenocarcinoma cell lines showed a range of sensitivity to the RNR inhibitors triapine (Fig. 2A), gemcitabine (Fig. 2B), and TAS1553 (Supplementary Fig. S4B) across all types of oncogene-driven lung adenocarcinoma models. As an example, we report here the efficacy of triapine on the *KRAS*^MUT^ H358 cell line. Triapine treatment (1.5 μmol/L for 72 hours) appreciably reduced the proliferation of the *KRAS*^MUT^ H358 cell line (Supplementary Fig. S4C).

To account for off-target effects of triapine, we performed CRISPR-mediated knockdown (Supplementary Fig. S4D) and overexpression (Supplementary Fig. S4D) of *RRM1* in the H358 model. CRISPR-mediated knockdown of *RRM1* significantly reduced cell viability in the H358 lung adenocarcinoma model (Fig. 2C and D), whereas *RRM1* overexpression was able to rescue the decreased viability in this model (Fig. 2C and D).

Given the role in DNA synthesis, we tried to elucidate the mechanism of action surrounding the ribonucleotide reductase inhibition (RNRi) effect. We first examined the impact of triapine on cell-cycle progression as a surrogate of cancer cell proliferation. Triapine treatment (1.5 μmol/L, 72 hours) led to an appreciable increase in G_2_–M arrest and an increase in the sub-G_0_ populations following treatment (Fig. 2E–G), supporting cell-cycle arrest as the mechanism of action of RNRi. The variability observed in other cell-cycle phases reflects intrinsic differences among the cell lines used. H358, PC9, and A925L cells possess distinct oncogenic and checkpoint-relevant mutational backgrounds that shape their cell-cycle responses to replication stress. H358 cells harbor *KRAS*^G12C^ with *TP53* loss, impairing canonical DNA damage checkpoints; PC9 cells are driven by an *EGFR*^exon19^ deletion that enforces strong G_1_–S checkpoint control; and A925L represents a molecularly distinct lung adenocarcinoma context (Supplementary Table S8). Accordingly, although RNR inhibition consistently increases the G_0_ population across all models, variability in the distribution of G_1_-, S-, and G_2_–M-phase reflects cell line–specific differences in oncogene-driven checkpoint engagement rather than inconsistent treatment response.

Moreover, in the NSCLC real-world cohort, DNA damage pathways were among the top pathways associated with high *RRM1* and *RRM2* expression, confirming the involvement of RNR in the DNA damage response (Fig. 2H). The cell-cycle arrest induced by RNR inhibition could then occur through impairment of the DNA damage response in a highly proliferative context.

These findings establish *RRM1* and *RRM2* as critical therapeutic targets in NSCLC independently of oncogenic drivers, with pharmacologic RNR inhibition effectively suppressing proliferation and inducing cell-cycle arrest. Our results provide a strong rationale for further clinical evaluation of RNR-targeting strategies in NSCLC treatment.

### RNR inhibition causes DNA damage and replication stress in oncogene-driven lung adenocarcinoma

Given our findings that pharmacologic RNR inhibition induces G_2_–M arrest in oncogene-driven lung adenocarcinoma cells and that the DNA damage signature is highly associated with RNR gene expression in NSCLC, we next sought to determine whether the cell-cycle arrest induced by RNRi is associated with DNA damage and replication stress.

**Figure 3.**
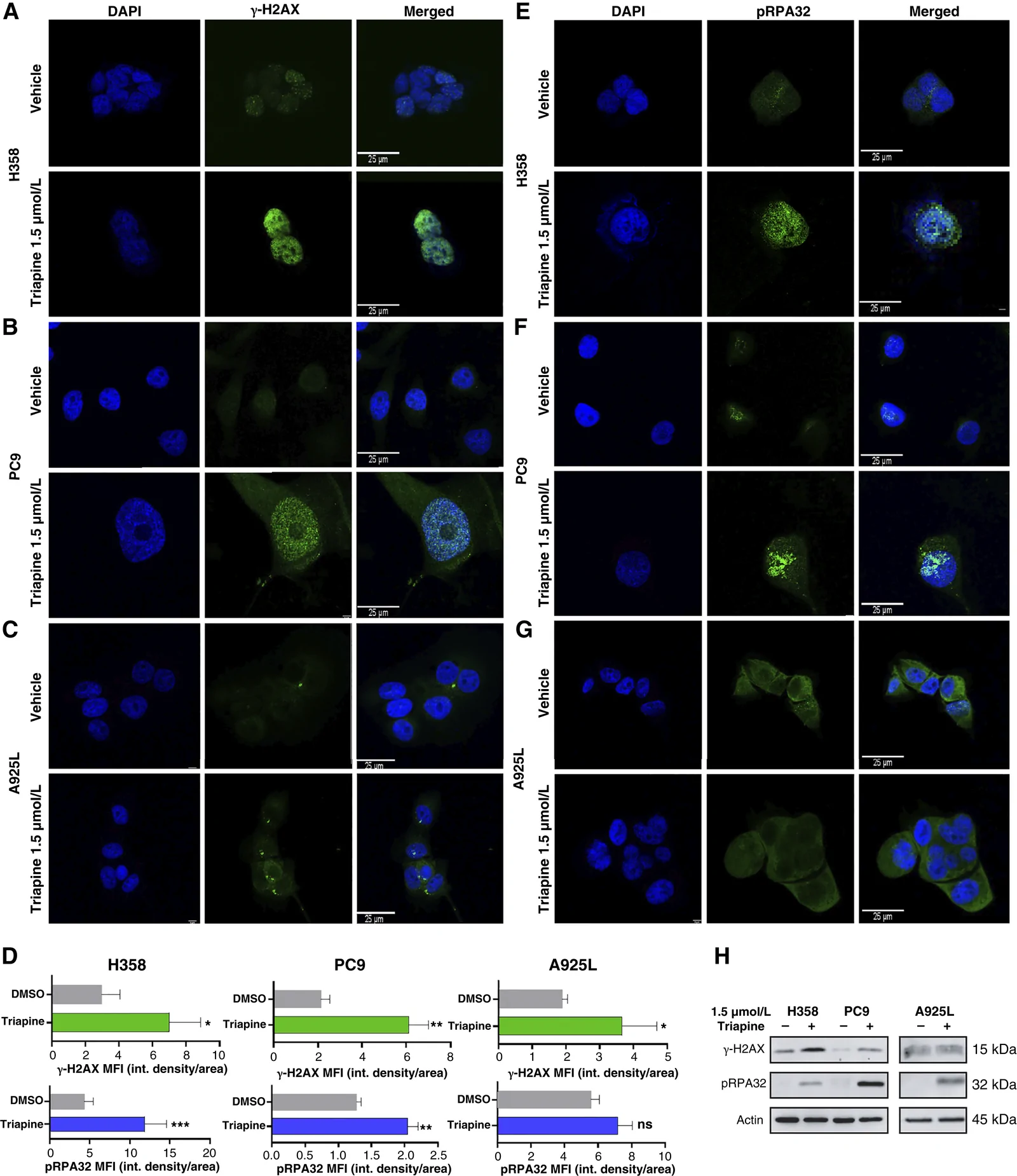
RNR inhibition causes DNA damage and replication stress in oncogene-driven lung adenocarcinomas. **A,** Representative IF staining images for H358 cells, stained with γ-H2AX and DAPI, −/+ triapine treatment (1.5 μmol/L, 72 hours). **B,** Representative IF staining images for PC9 cells, stained with γ-H2AX and DAPI, −/+ triapine treatment (1.5 μmol/L, 72 hours). **C,** Representative IF staining images for A925L cells, stained with γ-H2AX and DAPI, −/+ triapine treatment (1.5 μmol/L, 72 hours). **D,** Bar graph representation of the quantification of γ-H2AX and phospho-RPA32 mean fluorescence intensity (MFI) in different lung adenocarcinoma cell lines after triapine treatment. **E,** Representative IF staining images for H358 cells, stained with pRPA32 and DAPI, −/+ triapine treatment (1.5 μmol/L, 72 hours). **F,** Representative IF figure panel of PC9 cells stained with pRPA32, −/+ triapine treatment (1.5 μmol/L, 72 hours), and with phospho-RPA32 and DAPI. **G,** Representative IF staining images for A925L cells, stained with pRPA32 and DAPI, −/+ triapine treatment (1.5 μmol/L, 72 hours). **H,** Representative Western blot panel for γ-H2AX and phospho-RPA32 expression in PC9 and A925L cells upon triapine treatment (1.5 μmol/L, 72 hours). For comparisons among multiple groups, one-way analysis of variance (ANOVA) was performed; between two groups, a Student *t* test followed by Dunnett *post hoc* test was performed. *, *P* < 0.05; **, *P* < 0.01; ***, *P* < 0.001 were considered significant [*P* > 0.05 was considered nonsignificant (ns)]. The error bar represents the ± standard error of the mean (SEM), and the results are a representation of *n* = either 2 or 3 based on the experiments.

To assess DNA damage, we performed IF staining for phosphorylated histone H2AX (γ-H2AX)—a key marker of DNA double-strand breaks—on cell lines treated with RNRi triapine (1.5 μmol/L, 72 hours), which significantly increased γ-H2AX levels in the PC9, H358, and A925L lung adenocarcinoma cell lines (Fig. 3A–D). As replication stress often precedes DNA damage, we next examined the phosphorylation status of RPA32, a well-known replication stress marker. IF analysis showed increased phospho-RPA32 expression across all lung adenocarcinoma models following triapine treatment (1.5 μmol/L, 72 hours; Fig. 3D–G). Western blot analysis further confirmed the upregulation of γ-H2AX and phospho-RPA32 in triapine-treated PC9, H358, and A925L lung adenocarcinoma cell lines (Fig. 3H), reinforcing the role of RNR inhibition in DNA damage and replication stress induction.

### RNR inhibition induces ferroptotic cell death in oncogene-driven lung adenocarcinoma

**Figure 4.**
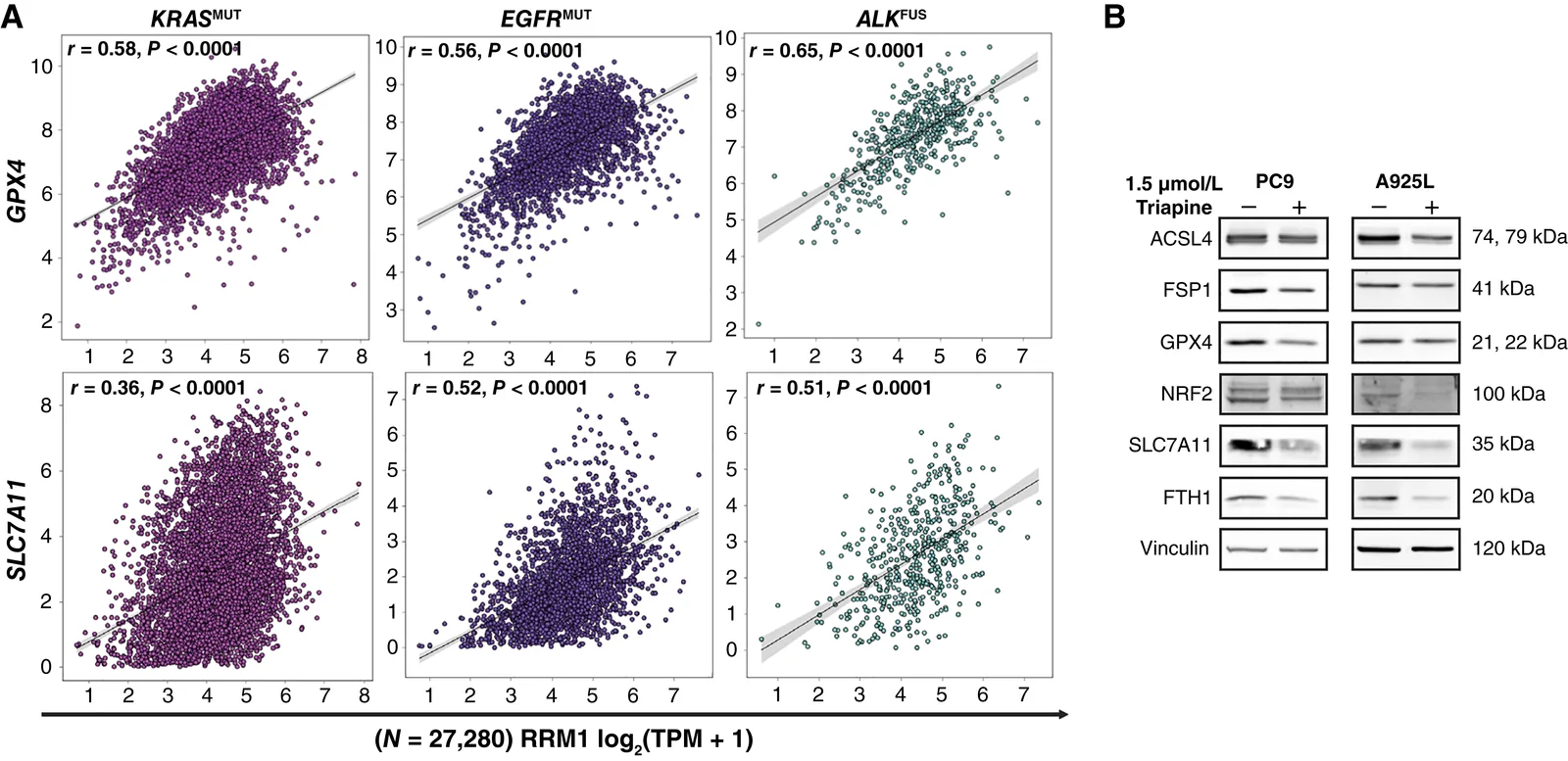
RNR inhibition modulates ferroptosis markers in NSCLC. **A,** Scatter plot representing the coexpression correlation between RRM1 and SLC7A11 and GPX4 in clinical samples with *KRAS*, *EGFR*, and *ALK* driver mutations. **B,** Representative Western blot panel for ferroptosis markers ACSL4, FSP1, GPX4, NRF2, SLC7A11, and FTH1 in PC9 and A925L cells upon triapine treatment (1.5 μmol/L, 72 hours). For comparisons among multiple groups, one-way analysis of variance (ANOVA) was performed; between two groups, a Student *t* test followed by Dunnett *post hoc* test was performed. *P* < 0.001 were considered significant [*P* > 0.05 was considered nonsignificant (ns)]. The error bar represents the ± standard error of the mean (SEM), and the results are a representation of *n* = either 2 or 3 based on the experiments.

When a treatment impairs the DNA damage response, replication stress and ROS accumulation increase, which may sensitize cells to ferroptosis. Investigating ferroptosis in the context of RNR inhibition could reveal off-target effects of this class of agents. A real-world cohort of NSCLC revealed a modest but significant correlation between the ferroptosis negative regulators *GPX4* and *SLC7A11*, and *RRM1/RRM2* expression in oncogene-driven samples (Fig. 4A; Supplementary Fig. S5A). These findings suggest that RNR inhibition may not only induce replication stress and DNA damage but also impair ferroptosis resistance mechanisms.

For that reason, we measured the amount of SLC7A11 (xCT) following RNR inhibition. SLC7A11 functions as a cystine/glutamate antiporter and plays a crucial role in ferroptosis resistance. Ferroptosis, a form of nonapoptotic cell death driven by lipid peroxidation, has emerged as a key vulnerability in cancer therapy. Previous work from others and our group (12, 20) has demonstrated that SLC7A11 depletion sensitizes cancer cells to ferroptosis. Interestingly, we identified a decrease in SLC7A11 (xCT) following RNR inhibition by Western blot (Fig. 4B) in both PC9 and A925L and in H358 cells (Supplementary Fig. S5B). We also assessed GPX4, ACSL4, NRF2, and FSP1, FTH1 expression by Western blot and evaluated iron dependency through FTH1 and TFRC expression (Fig. 4B; Supplementary Fig. S5B and S5C).

To further define the relationship between RNR expression and ferroptotic susceptibility, we assessed the correlation between RRM1/RRM2 expression and established ferroptosis-related markers, including ACSL4, SCD1, NRF2, and TFRC. Across all markers examined, RRM1 and RRM2 expression showed consistent positive correlations (Supplementary Fig. S5D), reinforcing a coordinated regulatory axis between the RNR complex and ferroptosis-associated pathways. These data strengthen the mechanistic rationale that RNR inhibition may shift NSCLC cells toward a ferroptosis-permissive state.

We next evaluated whether RNR suppression engages additional programmed cell death mechanisms. Analysis of apoptosis, autophagy, and necroptosis revealed that apoptosis is the dominant terminal pathway following RNR inhibition, as evidenced by robust cleaved PARP induction (Supplementary Fig. S5E–S5G). Although ferroptosis-related signaling is a primary contributor, apoptosis emerges as an additional cell death endpoint in NSCLC following RNR inhibition.

**Figure 5.**
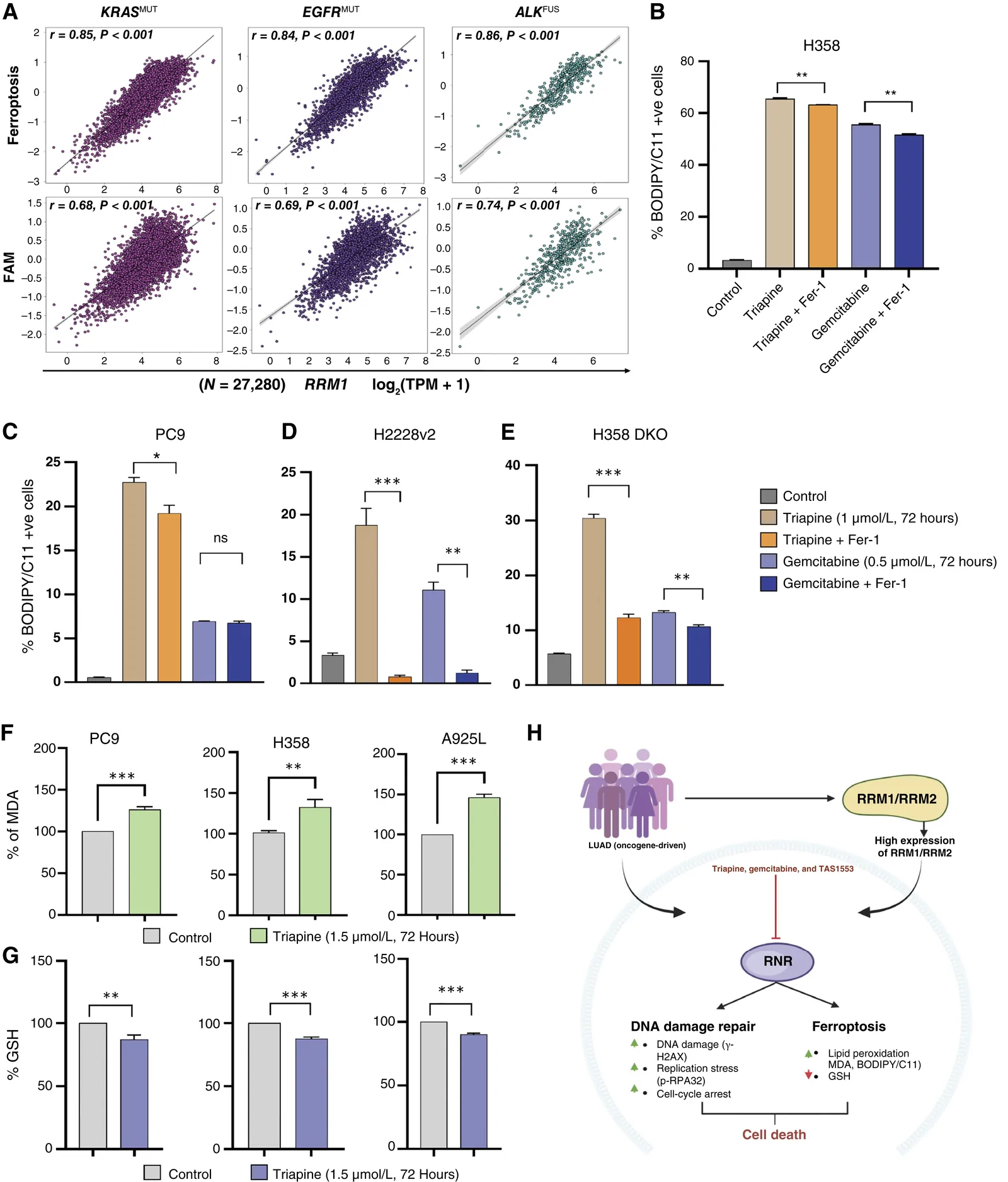
RNR inhibition induces ferroptosis in oncogene-driven lung adenocarcinoma cells by increasing lipid peroxidation. **A,** Scatter plot representation of correlation analysis of *RRM1* and ferroptosis signature gene set or RRM1 and fatty acid metabolism (FAM) signature gene sets. **B–E,** Bar graph representation of flow cytometry–based BODIPY/C11 staining for lipid peroxidation readout after triapine treatment (1 μmol/L, 72 hours) and gemcitabine (0.5 μmol/L, 72 hours) treatment with and without ferroptosis inhibitor, Fer-1, treatment on different lung adenocarcinoma cells. **F,** Bar graph representation of thiobarbituric acid reactive substance assay for measuring cellular MDA levels upon triapine treatment (1.5 μmol/L, 72 hours) in PC9, H358, and A925L cells. **G,** Bar graph representation of GSH assay for measuring cellular GSH levels upon triapine treatment (1.5 μmol/L, 72 hours) in PC9, H358, and A925L cells. **H,** Graphical representation of the effect of RNR inhibition in lung adenocarcinoma cells. For comparisons among multiple groups, one-way analysis of variance (ANOVA) was performed; between two groups, a Student *t* test followed by Dunnett *post hoc* test was performed. *, *P* < 0.05; **, *P* < 0.01; ***, *P* < 0.001 were considered significant [*P* > 0.05 was considered nonsignificant (ns)]. The error bar represents the ± standard error of the mean (SEM), and the results are a representation of *n* = either 2 or 3 based on the experiments.

Given our findings that RNR inhibition downregulates SLC7A11, GPX4, and critical ferroptosis regulators, we investigated whether targeting *RRM1*/*RRM2* induces ferroptotic cell death in lung adenocarcinoma. Analysis of NSCLC clinical samples demonstrated a significant positive correlation between *RRM1* (Fig. 5A) and *RRM2* (Supplementary Fig. S6A) expression with the ferroptosis gene signature and fatty acid metabolism pathway genes (Supplementary Table S9) in oncogene-driven lung adenocarcinoma, supporting a potential connection between RNR activity and ferroptotic cell death.

To directly assess ferroptosis induction, we performed a BODIPY/C11 lipid peroxidation assay in lung adenocarcinoma cell lines treated with triapine (1 μmol/L, 72 hours) or gemcitabine (500 nmol/L, 72 hours). Both treatments significantly increased BODIPY/C11-positive cells in all lung adenocarcinoma models compared with controls, which was reversed by Fer-1 treatment, a ferroptosis inhibitor (Fig. 5B–D). Notably, triapine treatment also led to a marked increase in lipid peroxidation in the ferroptosis-resistant *KRAS*^MUT^ model with *STK11/KEAP1* comutation (H358 double knock-out; Fig. 5E; refs. 12, 20).

MDA accumulation, a marker of lipid peroxidation, was significantly increased in all lung adenocarcinoma models following triapine treatment (Fig. 5F). Additionally, intracellular GSH levels, which counteract oxidative stress and ferroptosis, were markedly depleted upon RNR inhibition with triapine treatment (Fig. 5G). The reduction in GSH further supports our assertion that ferroptotic cell death is driven by *RRM1*/*RRM2* inhibition.

These findings establish that pharmacologic RNR inhibition promotes ferroptotic cell death in oncogene-driven lung adenocarcinoma by inducing lipid peroxidation and depleting GSH. Targeting *RRM1*/*RRM2* with triapine offers a promising therapeutic avenue to exploit ferroptosis as a cancer vulnerability in this setting (Fig. 5H). These findings establish that RNR inhibition induces DNA damage and replication stress and reduces ferroptosis resistance in oncogene-driven lung adenocarcinoma. This suggests that targeting *RRM1*/*RRM2* could enhance cancer cell vulnerability through both DNA damage repair and ferroptosis sensitization, warranting further investigation into the molecular underpinnings of this mechanism.

## Discussion

Our study underscores the therapeutic potential of targeting RNR by inhibiting its key components, *RRM1* and *RRM2*, in oncogene-driven lung adenocarcinoma. We primarily demonstrated that the driver mutations in *KRAS*, *EGFR*, *ALK*, *MET*^ex14^, *RET*^+^, and *ROS*^+^ genes exhibited elevated levels of *RRM1* and *RRM2* when associated with *TP53* molecular alteration, suggesting a high dependency on nucleotide metabolism in *TP53*-mutated lung adenocarcinoma (25–27). This observation aligns with previous reports and corroborates data showing that dysregulated nucleotide biosynthesis is linked to aggressive tumor behavior and poor prognosis in lung adenocarcinoma (28, 29). A key finding from our study is the strong correlation between *RRM1* and *RRM2* expression across lung adenocarcinoma clinical samples, irrespective of driver mutation. Our data further establish that high *RRM1*/*RRM2* expression is associated with significantly poorer survival outcomes, reinforcing their prognostic value.

Previous reports have shown that targeting RNR causes G_2_–M cell-cycle arrest in cancer cells (30, 31). Similarly, we have observed a substantial suppression of lung adenocarcinoma cell proliferation, followed by the induction of G_2_–M cell-cycle arrest and increased markers for DNA damage (γ-H2AX) and replication stress (phospho-RPA32) upon RNR inhibition. Additionally, our study reveals that RNR inhibition leads to the depletion of GPX4 and SLC7A11, key regulators of ferroptosis resistance, and other markers like NRF2, ACSL4, FTH-1, and TFRC were assessed. RNR inhibition leads to the depletion of GPX4 and SLC7A11, key regulators of ferroptosis resistance, alongside the assessment of additional markers, including NRF2, ACSL4, FTH1, and TFRC. Notably, ACSL4 abundance is reduced despite its established role in promoting ferroptosis. However, ACSL4-independent ferroptosis has been described (32), indicating that ferroptotic execution can proceed through alternative, GPX4-centered mechanisms. Accordingly, the role of ACSL4 in RNR inhibition-mediated ferroptosis in oncogene-driven NSCLC warrants further investigation. This further suggests that targeting *RRM1* and *RRM2* may sensitize lung adenocarcinoma cells to ferroptotic cell death driven by lipid peroxidation (22, 33). RNR inhibitors have previously been explored in various cancer models (17, 34, 35), but their impact on ferroptosis susceptibility in lung adenocarcinoma was not well characterized (36, 37). Our study advances mechanistic insights and links among RNR inhibition, replication stress, and ferroptotic cell death, complementing work that connects nucleotide metabolism disruption with oxidative stress vulnerability (36).

Notably, our study demonstrates the efficacy of RNR inhibition across major oncogene-driven lung adenocarcinoma models (*KRAS*, *EGFR*, *ALK*, *MET*^ex14^, *ROS*, and *RET* mutants), indicating a potentially broad vulnerability of the RNR pathway in this setting. Additionally, our study provides compelling real-world data and *in vitro* evidence that support RNR inhibition as a feasible therapeutic strategy. Although clinical trials investigating RNR inhibitors have included patients with pretreated NSCLC with oncogene-driven tumors, no specific data are available about the efficacy of RNR inhibition in this molecular subset. Given the potential vulnerability of these oncogene-addicted tumors to RNR targeting, combining RNR inhibitors with standard or targeted therapies represents a promising strategy that could be explored in future clinical trial designs. However, we emphasize the need for preclinical studies to confirm the efficacy of our approach and safety features first. Although our data suggest a link between RRM1/2 activity and ferroptosis sensitivity, the mechanistic basis of this relationship was not delineated in this study. Future work will be required to define the specific pathways through which the RNR complex influences ferroptotic regulation in lung adenocarcinoma. Moreover, the observation of alternate forms of cell death should be evaluated to determine the temporal relationship between RNR inhibition and ferroptosis. Although RNR has been linked to maintaining mitochondrial DNA copy number and mitochondrial permeability in different cancer types (38–40), the effect of RNR inhibition on mitochondrial function of our NSCLC preclinical models needs further investigation and clinical profiling. To further the translational potential of our findings, studies are required to assess the therapeutic impact of RNR inhibitors in lung adenocarcinoma mouse models and potential combination strategies that target oxidative stress pathways to enhance treatment efficacy in oncogene-driven settings.

Our findings establish RRM1 and RRM2 as prognostic biomarkers and potential therapeutic targets in mutation-driven lung adenocarcinoma. Bioinformatic analysis of a large scale lung adenocarcinoma cohort (*N* = 27,280) reveals key insights into the oncogenic contribution of RNR activity. These real-world results not only show that pharmacologic inhibition of RNR is a viable clinical approach but also that RNR expression can be used as an indicator of prognosis for specific oncogenic drivers of NSCLC, going beyond previous studies that lacked the sample size for such delineations. In addition, we validated that susceptibility to ferroptosis can be induced via RNR inhibition with both bioinformatic analysis and *in vitro* experiments. These results reveal the potential for a mechanistic and transcriptional role of RNRs promoting ferroptosis resistance in NSCLC when a direct correlation has yet to be reported. Pharmacologic RNR inhibitors, including triapine, which are already being tested in clinical trials, can offer a promising strategy for patients with specific genetic alterations by inducing replication stress, DNA damage, and ferroptosis. Furthermore, research in preclinical models is essential to translate these findings into clinical applications.

## Supplementary Material

Supplementary Figure S1 RRM1 and RRM2 expression in NSCLC clinical samples

Supplementary Figure S2 Stage progression is the primary driver of increased risk of death, independent of RRM1 expression or driver mutation status.

Supplementary Figure S3 Stage progression is the primary driver of increased risk of death, independent of RRM2 expression or driver mutation status.

Supplementary Figure S4 Genetic inhibition of RNR decreases cell proliferation, which is rescued by RNR re-expression

Supplementary Figure S5 RRM1 and RRM2 expressions show a positive correlation with ferroptosis markers in NSCLC clinical samples

Supplementary Figure S6 Correlation of RRM2 with ferroptosis and fatty acid metabolism signature in NSCLC clinical samples

Supplementary Table S1 Patient Demographics

Supplementary Table S2 List of the drugs/small molecule inhibitors

Supplementary Table S3 List of the Cell Lines used

Supplementary Table S4 List of the Critical Commercial Assays/Kits/reagents

Supplementary Table S5 List of Antibodies used

Supplementary Table S6 RRM1 and RRM2 expression (TPM+1) in LUAD samples (TP53wt vs TP53mut)

Supplementary Table S7 RRM1 and RRM2 expression (TPM+1) in LUAD samples among the key driver mutations

Supplementary Table S8 Mutation Profile of NSCLC Cell Lines used in the study

Supplementary Table S9 Gene Signature Lists – Ferroptosis and Fatty Acid Metabolism

## Data Availability

All raw data generated in this study and the datasets analyzed during the current study are available from the corresponding author upon request. The deidentified sequencing data are owned by Caris Life Sciences, and qualified researchers can apply for access to these summarized data by signing a data usage agreement. The data supporting this study’s findings are available from the TCGA PanCancer Atlas of Lung Adenocarcinoma at cBioPortal.
