# Supplementary material for: Ribonucleotide Reductase Inhibition Triggers Ferroptosis in Genetically Defined Subsets of Non–Small Cell Lung Cancer
Source: Cancer Res Commun. 2026 Sep 11;6(9):2136–48. doi: 10.1158/2767-9764.CRC-25-0829 (PMC13564884; doi:10.1158/2767-9764.CRC-25-0829)
Supplement: Supplementary file 1 [file crc-25-0829_supplementary_figure_s1_suppsf1.docx]

**Supplementary Figure S1. *RRM1* and *RRM2* expression in NSCLC clinical samples**

**A)** Scatter plot showing the correlation between *RRM1* and *RRM2* expression in NSCLC clinical samples. **B-D)** Scatter plots showing the correlation between *RRM1* and *RRM2* expression in *KRAS, EGFR*, and *ALK* mutant LUAD clinical samples. **E)** Representative violin plots of *RRM1* and *RRM2* expression in the TCGA LUAD dataset comparing *TP53wt* and *TP53mut* samples. **F)** Kaplan-Meier plot stratified by *RRM1* expression in the *METex14* mutant cohort. **G-I)** Kaplan-Meier plots stratified by *RRM2* expression in the *METex14, RET+,* and *ROS+* mutant cohorts. Chi-square, Fisher exact, Mann-Whitney U, and t tests with Welch correction were used, with adjustment for multiple hypothesis testing using the Benjamini-Hochberg method where appropriate. Statistical significance was defined as q<0.05.
