# Supplementary material for: Ribonucleotide Reductase Inhibition Triggers Ferroptosis in Genetically Defined Subsets of Non–Small Cell Lung Cancer
Source: Cancer Res Commun. 2026 Sep 11;6(9):2136–48. doi: 10.1158/2767-9764.CRC-25-0829 (PMC13564884; doi:10.1158/2767-9764.CRC-25-0829)
Supplement: Supplementary file 2 [file crc-25-0829_supplementary_figure_s2_suppsf2.docx]

**Supplementary Figure S2: Stage progression is the primary driver of increased risk of death, independent of *RRM1* expression or driver mutation status.**

In TCGA-LUAD dataset: A-C) Forest plots for overall survival (OS) and progression-free survival (PFS) were generated using models that included *RRM1* gene expression, disease stage, and *KRAS* (A), *EGFR* (B), or *ALK* (C) mutation status. For comparisons among multiple groups, one-way analysis of variance (ANOVA) was performed; for comparisons between two groups, a Student t-test followed by Dunnett’s post-hoc test was performed. *P<0.05 (*), P<0.01 (**), and P<0.001 (***)* were considered significant, while P>0.05 was considered non-significant (ns). For survival and progression-free analyses, a multivariate Cox proportional hazards regression model.
