# Supplementary material for: Ribonucleotide Reductase Inhibition Triggers Ferroptosis in Genetically Defined Subsets of Non–Small Cell Lung Cancer
Source: Cancer Res Commun. 2026 Sep 11;6(9):2136–48. doi: 10.1158/2767-9764.CRC-25-0829 (PMC13564884; doi:10.1158/2767-9764.CRC-25-0829)
Supplement: Supplementary file 4 [file crc-25-0829_supplementary_figure_s4_suppsf4.docx]

**Supplementary Figure S4**

**Supplementary Figure S4. Genetic inhibition of RNR decreases cell proliferation, which is rescued by RNR re-expression**

**A)** Bar graph representing the IC50 values of TAS1553 in LUAD cell lines. **B)** Dot plot presentation of reanalyzed CRISPR screen data (12). **C)** Crystal violet staining of H358 cells and different phenotypes (20) following triapine treatment (1.5 uM, 72H). **D)** Representative western blot images showing RRM1 expression after CRISPR-mediated genetic knockdown and genetic overexpression of *RRM1* in H358 cells. For comparisons among multiple groups, one-way analysis of variance (ANOVA) was performed; for comparisons between two groups, a Student t-test followed by Dunnett’s post-hoc test was performed. *P*<0.05 (*), *P*<0.01 (**), and *P*<0.001 (***) were considered significant, while P>0.05 was considered non-significant (ns). The error bars represent ± standard error of the mean (SEM), and the results represent n = either 2 or 3, depending on the experiments.
