# Supplementary material for: Ribonucleotide Reductase Inhibition Triggers Ferroptosis in Genetically Defined Subsets of Non–Small Cell Lung Cancer
Source: Cancer Res Commun. 2026 Sep 11;6(9):2136–48. doi: 10.1158/2767-9764.CRC-25-0829 (PMC13564884; doi:10.1158/2767-9764.CRC-25-0829)
Supplement: Supplementary file 5 [file crc-25-0829_supplementary_figure_s5_suppsf5.docx]

**RRM1 and RRM2 expressions show a positive correlation with ferroptosis markers in NSCLC clinical samples**

**A)** Scatter plot showing the expression correlation between *RRM2* and *GPX4* and *SLC7A11* in NSCLC clinical samples with *KRAS, EGFR,* and *ALK* driver mutations. **B)** Representative western blot panel for ferroptosis markers NRF2, SLC7A11, and FTH1 in H358 cells following triapine treatment (1.5 uM, 72 hours). **C)** Representative western blot panel and densitometric analysis for the ferroptosis marker TFRC in PC9 and A925L cells following triapine treatment (1.5 uM, 72 hours). **D)** Scatter plot showing the expression correlation between *RRM1* and *ACSL4, NRF2, SCD1*, and *TFRC,* and similarly, the correlation between *RRM2* and *ACSL4, NRF2, SCD1,* and *TFRC* in LUAD clinical samples. **E)** Representative western blot panel for the apoptosis marker cleaved PARP in H358, PC9, and A925L cells following triapine treatment (1.5 uM, 72 hours). **F)** Representative western blot panel and densitometry analysis for the autophagy marker LC3A/B in H358, PC9, and A925L cells following triapine treatment (1.5 uM, 72 hours). **G)** Representative Western blot panel for the necroptosis marker phospho-RIP3 in H358, PC9, and A925L cells following triapine treatment (1.5 uM, 72 hours).
