# Supplementary material for: Ribonucleotide Reductase Inhibition Triggers Ferroptosis in Genetically Defined Subsets of Non–Small Cell Lung Cancer
Source: Cancer Res Commun. 2026 Sep 11;6(9):2136–48. doi: 10.1158/2767-9764.CRC-25-0829 (PMC13564884; doi:10.1158/2767-9764.CRC-25-0829)
Supplement: Supplementary file 6 [file crc-25-0829_supplementary_figure_s6_suppsf6.docx]

**Supplementary Figure S6**

**Supplementary Figure S6. Correlation of RRM2 with ferroptosis and fatty acid metabolism signature in NSCLC clinical samples**

**A)** Scatter plot showing the expression correlation between *RRM2* and the ferroptosis signature gene set in NSCLC clinical samples with *KRAS, EGFR*, and *ALK* driver mutations. **B)** Scatter plot showing the co-expression correlation between *RRM2* and the fatty acid metabolism gene set in NSCLC clinical samples with *KRAS, EGFR,* and *ALK* driver mutations.
