# Supplementary material for: Ribonucleotide Reductase Inhibition Triggers Ferroptosis in Genetically Defined Subsets of Non–Small Cell Lung Cancer
Source: Cancer Res Commun. 2026 Sep 11;6(9):2136–48. doi: 10.1158/2767-9764.CRC-25-0829 (PMC13564884; doi:10.1158/2767-9764.CRC-25-0829)
Supplement: Supplementary file 7 [file crc-25-0829_supplementary_table_s2_suppst2.docx]

| **Supplementary Table S2: List of the drugs / small molecule inhibitors** | | |
| --- | --- | --- |
| Ferrostatin-1 | Selleckchem | Cat# S7243 |
| Triapine | Selleckchem | Cat# S7470 |
| Gemcitabine | Selleckchem | Cat# S1714 |
| TAS-1553 | Medchemexpress (MCE) | Cat# HY-150785 |
