# Supplementary material for: Ribonucleotide Reductase Inhibition Triggers Ferroptosis in Genetically Defined Subsets of Non–Small Cell Lung Cancer
Source: Cancer Res Commun. 2026 Sep 11;6(9):2136–48. doi: 10.1158/2767-9764.CRC-25-0829 (PMC13564884; doi:10.1158/2767-9764.CRC-25-0829)
Supplement: Supplementary file 8 [file crc-25-0829_supplementary_table_s3_suppst3.docx]

|  |  |  |  |  |  |
| --- | --- | --- | --- | --- | --- |
|  |  |  |  |  |  |
|  |  |  |  |  |  |
|  |  |  |  |  |  |

| **Supplementary Table S3: List of the Cell Lines used** | | |
| --- | --- | --- |
| **Reagent/Resource** | **Source** | **Identifier#** |
| **NCI-H358** | ATCC | CVCL_1559 |
| **NCI-H2228v2** | ATCC | CVCL_1543 |
| **PC9** | ATCC | CVCL_B260 |
| **A925L** | ATCC | CVCL_U096 |
| **H23** | ATCC | CVCL_1547 |
| **HCC827** | ATCC | CVCL_2063 |
| **H358 STK11/ KEAP1 KO** | Wohlhieter et al., 2020 | (PMID: 33264619) |

`
