# Supplementary material for: Ribonucleotide Reductase Inhibition Triggers Ferroptosis in Genetically Defined Subsets of Non–Small Cell Lung Cancer
Source: Cancer Res Commun. 2026 Sep 11;6(9):2136–48. doi: 10.1158/2767-9764.CRC-25-0829 (PMC13564884; doi:10.1158/2767-9764.CRC-25-0829)
Supplement: Supplementary file 9 [file crc-25-0829_supplementary_table_s4_suppst4.docx]

| **Supplementary Table S4: List of the Critical Commercial Assays/Kits/reagents** | | |
| --- | --- | --- |
| **Reagent/Resource** | **Source** | **Identifier/ Cat#** |
| Trypan Blue | Thermo, Invitrogen | Cat# T10282 |
| Crystal Violet | Thermo, Invitrogen | Cat# B2193214 |
| BODIPY 581/591 C11 | Thermo, Invitrogen | Cat# D3861 |
| Pierce RIPA Buffer | Thermo, Invitrogen | Cat# 89900 |
| Protease inhibitor cocktail | Thermo, Invitrogen | Cat# A32955 |
| HALT Protease and Phosphatase Inhibitor (100X) | Therm, Invitrogen | Cat# 1861281 |
| Coomassie Protein Assay Reagent | Thermo, Invitrogen | Cat# 1856209 |
| Cell Titer Glo assay | Promega | Cat# G7571 |
| GSH Estimation Kit | Thermo, Invitrogen | Cat# EIAGSHC |
| Polybrene | Tocris, R&D systems | Cat# 7711 |
| TBARS assay kit | Cayman Chemical | Cat# 10009055 |
| Fx Cycle PI/RNAse staining solution | Thermo, Invitrogen | Cat# F10797 |
| Lamelli SDS sample buffer, reducing (4X) | Thermo, Invitrogen | Cat# J60015.AC |
