# Supplementary material for: Ribonucleotide Reductase Inhibition Triggers Ferroptosis in Genetically Defined Subsets of Non–Small Cell Lung Cancer
Source: Cancer Res Commun. 2026 Sep 11;6(9):2136–48. doi: 10.1158/2767-9764.CRC-25-0829 (PMC13564884; doi:10.1158/2767-9764.CRC-25-0829)
Supplement: Supplementary file 10 [file crc-25-0829_supplementary_table_s5_suppst5.docx]

| **Supplementary Table S5: List of Antibodies used** | | |
| --- | --- | --- |
| **Reagent/Resource** | **Source** | **Identifier/ Cat#** |
| Anti-SLC7A11 | Cell Signaling Technology | Cat# 12691, RRID: AB_2687474 |
| Anti β-Actin | Cell Signaling Technology | Cat# 4967, RRID: AB_330288 |
| Anti-Vinculin | Cell Signaling Technology | Cat# 4650, RRID: AB_10559207 |
| Phospho RPA32 | Cell Signaling Technology | Cat# 54762, RRID: AB_2799471 |
| Phospho Gamma H2AX | Cell Signaling Technology | Cat# 9718, RRID: AB_2118009 |
| Lamin B1 | Cell signaling Technology | Cat# 68591, RRID:AB_2799751 |
| Anti-RRM1 | Cell signaling Technology | Cat# 8637, RRID: AB11217623 |
| Anti-RRM2 | Abcam | Cat# ab172476, RRID: AB_3741628 |
| Anti-ACSL4 | Invitrogen | Cat# PA5-271173, RRID: AB_2544613 |
| Anti-FTH1 | Cell signaling Technology | Cat# 4393, RRID: AB_11217441 |
| Anti-GPX4 | Cell signaling Technology | Cat# 52455, RRID: AB_2924984 |
| Anti-FSP1 | Cell signaling Technology | Cat# 24972, RRID: AB_3090192 |
| Anti-LC3A/B | Cell signaling Technology | Cat# 12741, RRID: AB_2617131 |
| Anti-TFRC | Cell signaling Technology | Cat# 13208, RRID: AB_2798150 |
| Anti-phospho-RIP3 | Cell signaling Technology | Cat# 93654, RRID: AB_2800206 |
| Anti-RIP3 | Cell signaling Technology | Cat# 13526, RRID: AB_2687467 |
| Anti-Cleaved PARP | Cell Signaling Technology | Cat# 94885, RRID: AB_10699459 |
