# Supplementary material for: Ribonucleotide Reductase Inhibition Triggers Ferroptosis in Genetically Defined Subsets of Non–Small Cell Lung Cancer
Source: Cancer Res Commun. 2026 Sep 11;6(9):2136–48. doi: 10.1158/2767-9764.CRC-25-0829 (PMC13564884; doi:10.1158/2767-9764.CRC-25-0829)
Supplement: Supplementary file 13 [file crc-25-0829_supplementary_table_s8_suppst8.docx]

**Supplementary Table S8: Mutation Profile of NSCLC Cell Lines used in the study**

| **Cell lines** | ***ALK*** | ***EGFR*** | ***KRAS*** | ***TP53*** | ***STK11*** |
| --- | --- | --- | --- | --- | --- |
| **H358** | WT | WT | **G12C** | Null/Deletion | Mutant |
| **PC9** | WT | **Exon 19 del (E746-A750del)** | WT | Mutant | WT |
| **A925L** | **EML4-ALK fusion-positive** | WT | WT | Mutant | WT |
