# Supplementary material for: Ribonucleotide Reductase Inhibition Triggers Ferroptosis in Genetically Defined Subsets of Non–Small Cell Lung Cancer
Source: Cancer Res Commun. 2026 Sep 11;6(9):2136–48. doi: 10.1158/2767-9764.CRC-25-0829 (PMC13564884; doi:10.1158/2767-9764.CRC-25-0829)
Supplement: Supplementary file 14 [file crc-25-0829_supplementary_table_s9_suppst9.docx]

| **Supplementary Table S9: Gene Signature Lists – Ferroptosis and Fatty Acid Metabolism** | |
| --- | --- |
| **Gene Signature** | **Gene ID** |
| Ferroptosis | HSPB1 |
| Ferroptosis | PTGS2 |
| Ferroptosis | SLC1A4 |
| Ferroptosis | AIFM2 |
| Ferroptosis | ABCC1 |
| Ferroptosis | DDIT3 |
| Ferroptosis | DDIT4 |
| Ferroptosis | CA9 |
| Ferroptosis | HSPA5 |
| Ferroptosis | CHAC1 |
| Ferroptosis | TP53 |
| Ferroptosis | ATM |
| Ferroptosis | BECN1 |
| Ferroptosis | SIRT1 |
| Ferroptosis | RRM1 |
| Ferroptosis | RRM2 |
| Ferroptosis | DHODH |
| Fatty Acid Metabolism | SQLE |
| Fatty Acid Metabolism | PEBP1 |
| Fatty Acid Metabolism | SCD |
| Fatty Acid Metabolism | AKR1C1 |
| Fatty Acid Metabolism | AKR1C2 |
| Fatty Acid Metabolism | AKR1C3 |
| Fatty Acid Metabolism | ACSL4 |
| Fatty Acid Metabolism | ALOX12 |
| Fatty Acid Metabolism | ALOX12B |
| Fatty Acid Metabolism | ALOX15 |
| Fatty Acid Metabolism | ALOX15B |
| Fatty Acid Metabolism | ALOX5 |
| Fatty Acid Metabolism | ALOXE3 |
| Fatty Acid Metabolism | ATF4 |
| Fatty Acid Metabolism | BRD4 |
| Fatty Acid Metabolism | SIK1 |
| Fatty Acid Metabolism | PPARGC1A |
