# Supplementary material for: Ribonucleotide Reductase Inhibition Triggers Ferroptosis in Genetically Defined Subsets of Non–Small Cell Lung Cancer
Source: Cancer Res Commun. 2026 Sep 11;6(9):2136–48. doi: 10.1158/2767-9764.CRC-25-0829 (PMC13564884; doi:10.1158/2767-9764.CRC-25-0829)
Supplement: Supplementary file 15 [file crc-25-0829_supplementary_table_s6_suppst6.docx]

**Supplementary Table S6: *RRM1* and *RRM2* expression (TPM+1) in LUAD samples (*TP53*^wt^ vs *TP53*^mut^)**
